# An Exploration of the Role of Hospital Committees to Enhance Productivity

**DOI:** 10.5539/gjhs.v8n3p199

**Published:** 2015-08-06

**Authors:** Hassan Amirabadi zadeh, Mohammad Reza Maleki, Masoud Salehi, Soudabeh Watankhah

**Affiliations:** 1 School of Management and Medical Information Sciences, International Campus (IUMS-IC), Iran University of Medical Sciences, Tehran, Iran; 2 Department of Biostatistics, Faculty of Public Health, Iran University of Medical Sciences, Tehran, Iran; 3 Health Care Management Department, Faculty of Management and Medical Information Sciences, Iran University of Medical Sciences, Tehran, Iran

**Keywords:** hospital committee, management, productivity, team efficiency, team effectiveness

## Abstract

Productivity is the main concern of hospitals as organizations providing health services. As the role of hospital committees is increasing and their productivity and performance improvement is very important, the present study was performed to identify weaknesses and strengths of committee sessions. This analytical-descriptive study was conducted cross- sectional from January to April in 2012. Summary of 405 committee session's agendas related to 11 kinds of committees in 8 hospitals (out of 23 hospitals) of capital cities in 3 provinces of Sistan and Balouchestan, South Khorasan and Khorasan Razavi in Iran were extracted. Data was collected through a form and was analyzed by SPSS16 software using descriptive statistics and variance analysis and content analysis technique. This study showed that the number of hospital committee's sessions holding in 2012 was more than 2011. The differences between public and private hospitals in terms of the following subjects were significant (P-Value < 0.001). In terms of the number of selected policies, participants of the committees, and the duration of the sessions the public hospitals had better conditions. And regarding documentation process, feedback of decisions to personnel and the implementation of the formulated policies in the committees, private hospitals performed better. According to the results of this study, to improve the productivity of hospital committees, it is suggested to motivate senior, tactical and operational managers to appropriately participate in the committees and necessary planning for the committees in advance is mandatory.

## 1. Introduction

More than two decades, organizations want to decrease grow of health care costs and also health organizations want to decrease health per capita and because in some cases, this resulted in quality being sacrificed for cost, a lot of patients could not take reception and they were deprived of service accessibility right and selection right. So, we suggest that by created productivity increase we can decrease costs and prevent quality fall (Goldberg & Kosinski, 2011). One way for productivity increase in recent years for improvement of health system in the world is paying attention to new structure and widespread organizational changes (Axler & Bogart, 1997). Also in Iran, for increasing productivity in hospitals, structure reform has been done to a large number including hospital committees (changing Committees number from 14 to 11 in 2011).

For productivity improvement, presence of vital information is necessary. At present, hospitals bear high stress for productivity improvement by internal performance without sacrificing quality of care. One of the main reasons for stress increase is ignorance of current information about hospitals which is very useful source for processes and productivity reform (Payne & Williams, 1991). And now in hospitals, documenting the activities such as setting the agenda related to hospital committee sessions and sending a copy to higher levels for planning is much emphasized.

Also, according to limited sources and control of health care costs, nowadays all beneficiaries search different strategies for productivity of work force one of which is innovations designed to increase work force at hospitals (Hams, 1991). It is obvious for all personnel that one of hospital problems are related to budget deficit and managers should dominate on this problem by policies and programs of hospital committees. So, at hospitals we should use suitable strategies about cost control such as model of cost determination based on activities. In this model, resource costs are considered exactly according to implemented activities by list of sub activities of an activity and cost rate of every activity (Livens, Faun, & Kesteloot, 2003) and also with better information presentation, we can use hospital strategic programming from management system model of cost according to activity (ABM). By hospital services improvement and by help of health care presentation and by quality and productivity improvement we can lead to better management and control of current sources of hospital (Arid, 1996). Also, hospital committees should be more active in planning such as participating in setting strategic and operational programs in hospitals.

At implemented study in Faghihi hospital in which cost model was used, it was shown that recognized costs price by this model is remarkably different from current price tariffs at hospital and abundant amount of non-direct costs of current sources are not used suitably at hospitals (Rajabi & Dabiri, 2012). Another item related to current sources of hospital is human resource about which, budget common commission of health care organizations has recommended that medical members and personnel present effective solutions and mechanisms for screening and evaluation of related quality of services to patients and organizational performance improvement. Many health and therapy institutes meet their needs by creating organized committees (Tackett & Kent, 1994). For better use of human resources, managers search many solutions. For example, nurse managers and hospital managers for productivity and empowering of nurses want to create nurse council in which using a new approach for a successful strategy is proposal of rearranged engineering of functional council of nurse organization so as to increase values of organization and create excitement at personnel by productivity promotion (Gokenbach, 2007).

As we know, committees are tools for mixing distributed knowledge and abilities of various parts of organization at format of one active and integrated unit. Committees fortify another kind of power separation for people who are responsible for their jobs but cannot work solely as organizational units and because organizational responsibilities are beyond abilities of persons merely, using committees can be used as suitable method and prevent person dictatorship approach (Harder’s, Trumann, & Setoff, 1998). Committees are managers assistant arms and a place for cooperative management that use experts, help solve current problems and assist in policy.

In Iran before autumn of 2011, there were 14 committees at hospitals which had 5 percent scores of hospital evaluation that led to hospital grade level and from December of 2011, this committees at hospital budget designation (replacing hospital evaluation case) changed to 11 committees that three of them were new, 2 committees were mixed and 7 committees from the 14 previous committees were unchanged. These 11 committees were sent to universities by ministry in the book of “hospital budget designation standards in Iran” and this instructions is the base of hospital committees performance. These eleven committees include:1- quality improvement committee 2- drug and therapy committee 3- medical ethics committee 4- medical documents and information technology committee 5- technical protection and job health committee 6- committee of mother and baby safety, promotion of natural born and feeding with mother milk 7- environmental health committee 8- committee of hospital infection control 9- death and side effects of pathology and tissues committee 10- committee of crisis and disasters 11- blood transfer medical committee (Jafari et al., 2010). Duty of each of these 11 official hospital committees has been announced in regulation sent by ministry and they have different duties but hospital managers can set duties similar to these duties for each committee. Kinds of committees, committees responsibilities and the minimum period of holding committees are determined by the ministry. Monitoring Committees in the context of accreditation and routine visits are planned by health deputy experts and accreditation team but adding new committees, the number and kind of committee members, setting internal regulations of committees and choosing kinds of decisions is determined by hospital.

The boss of committee can reduce sessions intervals and invite anyone necessary to the committees. Each committee has fixed members who have notification of the chief of the hospital for a certain period (usually for one year) and if the members aren’t efficient, other members are appointed by the chief of the hospital.

In hospital committees, the issues and problems and future programs are transmitted to higher levels or committee secretary from hospital sections and units, are prioritizing by the secretary and proposed in the sessions according to their importance.

Committees don’t usually have an income for their members and yet make each kind of decisions for hospitals and sometimes members aren’t paid at all for their membership.

According to Gantt chart written for hospitals which announces the time of sessions which is usually for a six-month period, and this schedule is given to the members 2-3 days before holding sessions, issues to be proposed in sessions, time and place of sessions is announced to the members.

One of indicators for evaluating hospital performance is activity of committees because a part of evaluation scores and accreditation in hospitals is hospital committees. (5% of evaluation scores for identifying hospital level.) and one of the most important and measurable indicators is the number of held hospital sessions and according to regulation sent by ministry the interval of sessions is specified.

Indicators specified in Table 1 show the performance position of committees and show committees favorability. Importance of committees and team working is very significant especially about purchase committee that committees not only evaluate primary materials before purchase but also review current materials at hospital that leads to favorite quality, quantity, productivity cost at purchase amount and present materials at hospital (Boergadine, 1997). A study was done by Sajadi et al. in 2007 about the influence of productivity committees the results of which showed that in general the maximum influence of productivity committees related to the improvement of presented services at centers and at second level, committees establishment had influence on increase of income and satisfaction of customers and also influence on centers cost decrease was at third place (Sajjadi & Toghiani, 2011). Up to now, we have not observed comprehensive study about the position of holding hospital committees sessions in Iran; So, as a part of doctorate thesis in health services management, this study was done for recognizing the performance of hospital committee's sessions.

**Table 1:** Descriptive parameters of hospital committees sessions positions in 2011 and 2012

Row	Title	Year2011						Year2012					

		Number	Minimum	maximum	Total	Mean	Standard of deviation	number	Minimum	Maximum	Total	Mean	Standard of deviation
1	Held numbers	85	1	17	527	6.20	3.37	79	1	18	507	6.42	**2.93**
2	Sessions participators number	85	5	18	875	10.29	2.46	79	5	21	834	10.56	**2.65**
3	Presence number of committee chairman	85	0	11	304	3.58	2.64	79	0	12	328	4.15	**3.05**
4	Bill numbers of every session	85	1	18	437	5.14	3.50	79	1	13	393	4.97	**2.74**
5	Bills related to future programs of human resource	85	**0**	8	91	1.07	1.44	79	**0**	19	120	1.52	**2.61**
6	Bills related to future programs of financial resource	85	**0**	3	5	0.06	0.36	79	**0**	3	17	0.22	**0.59**
7	Bills related to future programs, process and methods	85	**0**	6	78	0.92	1.36	79	**0**	11	156	1.97	**2.43**
8	Bills related to current problems of human resource	85	**0**	8	113	1.33	1.64	79	**0**	6	110	1.39	**1.67**
9	Bills related to current problems of financial resource	85	**0**	13	206	2.42	3.30	79	**0**	27	332	4.20	**5.50**
10	Bills related to current problems, process and methods	85	**0**	24	343	4.04	4.77	79	**0**	24	427	5.41	**5.14**
11	Percent of attained bill goals	85	36	100	7229	85.05	15.20	79	30	100	6509	82.39	**14.68**
12	Lack of bill implementation because of lack of necessary attention	85	**0**	8	95	1.12	1.76	79	**0**	10	131	1.66	**2.12**
13	Lack of bill implementation because of lack of human resource shortage	85	**0**	4	34	0.40	0.79	79	**0**	3	28	0.35	**0.68**
14	Lack of bill implementation because of lack of expert analysis	85	**0**	5	37	0.44	0.79	79	**0**	9	67	0.85	**1.71**
15	Lack of bill implementation because of lack of experts cooperation	85	**0**	5	32	0.38	0.90	79	**0**	3	23	0.29	**0.72**
16	Lack of bill implementation because of lack of financial problems	84	**0**	4	31	0.37	0.88	79	**0**	5	65	0.82	**1.27**
17	Lack of bill implementation because of lack of other reasons	85	**0**	4	12	0.14	0.60	79	**0**	1	7	0.09	**0.29**

The aim of this article is to identify the structure and performance position of the 11 hospital committees in presence number of committee boss, sessions participants number, kinds of bills, rate of attained bill goals, reasons for lack of bill implementation and …. We hope that identifying strengths and weaknesses and presenting them to managers will cause committees performance improvement and as a result, hospital productivity improvement.

## 2. Methods

The present study is analytical-descriptive and it was conducted cross-sectional from January to April of 2012 for identifying the performance position of committees sessions in 8 hospitals among 23 existing private and governmental hospitals in the smallest and biggest hospitals (based on bed number) of centers of Sistan and Blouchestan, South Khorasan and Khorasan Razavi provinces in Iran. We chose these provinces because they were a sample of three financial statuses (good, intermediate and weak) in Iran.

Data is related to 2011 and 2012 agenda which was collected through the designed form after official correspondence and going to hospitals in 2012.

According to the conducted studies (Jackson & Olive, 2009; Kalaki, 2000; Rafiei, 1997) and existing regulations, there is a difference between the performance position of the biggest and the smallest governmental and private hospitals. So, we chose the smallest and biggest hospitals in order to better identify the kinds and causes of differences. On the other hand, because there wasn’t any private hospital in South Khorasan and Sistan and Balouchestan provinces, the private hospitals we studied belonged only to Khorasan Razavi province.

Because of time and cost limitations, two hospitals were selected from every province (governmental and private). Four hundred and five numbers of held hospital committee session agendas at 2011 and 2012 (according to Table 1) were entered to related form. Our inclusion and exclusion criteria for the study was in a way that all the agendas (typed and hand written) were accepted but verbal comments mentioned by committee chairperson was not accepted.

Measuring tool for evaluating performance was a self-made form for recording the performance position of hospital committees that following 17 variables were measurable and by comparing these variables in different hospitals, committee performance position was measurable.

For validity, some experts commented on the form and because all the information was registered, there was no need for verifying reliability.

For completing this form, at first with the presence of hospital committee secretary, we listed date of all held committee sessions and we recorded for every session, number of participants, name of session secretary, the number of bills of the session, type of bills (future plans or according to the existing problems including: problems of human resource, problems of financial resource, processes and methods), percent of implemented bills and the reasons for not implementing bills(because of the lack of necessary attention, lack of human resource, lack of expert analysis, lack of experts cooperation, lack of financial resources, other reasons). For sampling bills of every session for 2011 for every held committee session, we recorded 2 agendas one of them from first half of the year (spring season) and the other from second half of the year (winter season) and for 2012 for every committee 3 agenda related to first, second and third seasons were selected. Data related to every session bill including: bill name, implemented actions, percent of attained goals, reason of lack of bill implementation and bill kind (bill related to present problems or future programs) was recorded from archive of committees agenda into provided form.

Data was extracted from all existing agendas in hospitals -typed or handwritten-and only agendas related to these 11 committees were considered and other agendas related to committees out of list of these 11 committees were not included in the study.

The entered data was controlled by two persons and the differences were corrected. We spent about 3 hours for taking note of every agenda. Then, we entered data to SPSS 16 software and extracted following data. We studied way of data distribution and also at the rest using variance analysis tests, and Post Hoc tests, Multivariate tests, Mauchly^,^s tests of Sphericity, Tests of within-subject Effects, Tests of Between-Subject Effects and…., we compared average of various variables among relevant groups.

## 3. Results

Descriptive findings of present study were collected from 405 agenda from 8 hospitals out of 23 existing private and governmental hospitals in 3 provinces.

Data collected in this study and its results are presented in Table 1 based on the studied items.

Above table shows that:

-Average of held hospital committee sessions in 2011 and 2012 year was 6.20±3.37 and 6.42±2.93 that the maximum number of held sessions in 2012 was 18.

-Average number of participants in hospital committees sessions at 2011 and 2012 was 10.29±2.46 and 10.56± 2.65 that in some sessions, number of participants was 21.

-Average of bill number in 2011 was 5.14±3.50 and in 2012 it was 4.97±2.74 that in some held committees of 2011, the number of bills reached up to 18.

-Most bills at hospital committees in 2011 and 2012 were related to current problems related to processes and methods, financial sources and human resource in order.

Rate of chairman presence in committees was about 57. %.

The most reasons for not implementing bills were lack of follow-up and lack of expert analysis.

The most bills were related to solving current problems of existing methods and processes which requires restructuring and integration processing.

The lowest reason for not implementing bills has been financial problems although we had thought that financial problems are the main cause of not implementing bills.

It seems that in private hospitals because of the nature of hospital, the most emphasis has been on efficiency and in governmental hospitals the most emphasis has been on effectiveness.

In the studied hospitals in 2012, only 75% of committees were held according to the predicting program. This percent has been 57% in 2011and perhaps one of the reasons for decreasing the number of hospital committees by the ministry has been the high number of committees one of its results being not holding committee sessions according to the ministry regulation.

-Among the studied reasons for not implementing hospital committees bills, (not follow-up required, lack of human resources, not expert analysis of bills, lack of expert cooperation, financial problems) the most reasons was not expert analysis of bills one of its causes being a high number of bills in each session. (5 numbers). In this regard, forming sub-committees is offered.

This study showed that the average number of every session bills was 2 cases in private hospitals in 2011 and 6 cases in 2012.

**Diagram 1:**
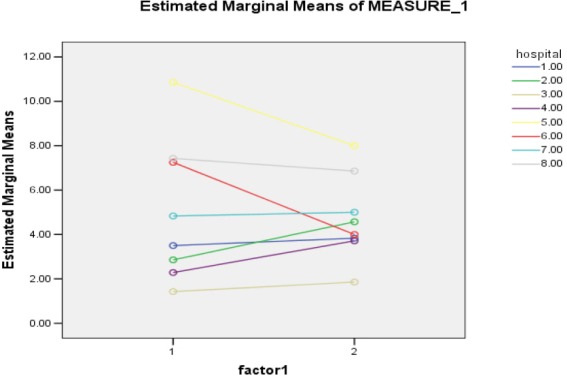
Average of bills in 2011 and 2012

As it is shown in Diagram 1, the comparison of committees performance in the studied hospitals for the variable “the number of held sessions” was done between 2011 and 2012.

The results of Duncan test in terms of hospitals performance position in committee sessions held in 2011 and unilateral variance analysis showed that held sessions of committees at 2011 had meaningful difference among hospitals statistically (p <0.001) but the differences between committees in each hospital was not meaningful. This observed meaningful difference is emanated from current difference in held committee sessions of hospitals 6, 2, 7, 8 and 4 as the least average number of held sessions and in hospitals 3 as the most average number with other hospitals (Diagram 1).

Also, repeated measurement analysis of the effect of time variable in 2011 year (first factor) and 2012 year (second factor) and hospital influence in variables of “average of participant members at committees”, “average of bills at committees”, “average of implemented bills” showed that average of this variables in different years among different hospitals has not meaningful difference from statistical aspect (P=0.208, P= 0.070) and (P=0.275, P=0.001) and P=0.497, P= 0.153). Eye-catching point related to meaningful bilateral influence of time and hospital from statistical view about variable of average of participating members in committees and average of bills in committees (p= 0.001 and p= 0.035). According to Table 1, we can observe that this bilateral influence of different hospitals behavior is the variable of bills in different years as we see that the average position of hospital numbers 5 and 6 in 2011 and 2012 show falling steep vector, the average position of hospital numbers 4 and 2 in 2011 and 2012 show rising steep vector and average position of hospital numbers 1 and 7in 2011 and 2012 show fixed steep vector. These changes are meaningful from statistical aspect.

About the size of committees, depending on the kind of committee, there are 5-21 members that in big hospitals the number of members is high (with the average of 14), but in small hospitals it is less (with the average of 9) and in private hospitals the number of members is much less than governmental hospitals (ratio of 7 to 12). The most number of members relates to “quality improvement committee” and “drug and therapy committee” and the lowest number relates to “medical ethics committee” and “medical documents and information technology committee”.

Time of sessions differs from 25 minutes to 90 minutes which is much less in private hospitals than governmental ones.

## 4. Discussion

Today committees are a manifestation of employee participation in decision-making and team working decreases errors and increases bills acceptability and their implementation.

About nature of committees, we can say that organizational structures and groups and teams performance are different from each other in entity and kind of decision-making. For example, interaction kind between team members, decision-making methods leading to final decision, interactions between management domain and personnel, personnel costs and present priorities at hospitals are done by committees. All of these items are proposed and verified at committees before implementation in hospital that it shows role of collective decision-making (Goodman, 1995). Also, the results of our study showed that the committee performance during the studied years, within hospitals and among different hospitals is different. A study showed that there were no significant statistical relationship between BOR and type of hospitals as educational or non-educational and also the location 9.of these hospitals in poor or rich areas. But BOR and hospital size measured by their number of active beds, had a positive significant relationship statistically. So, we can say that the kind and number of committees can be similar in educational or non-educational hospitals whether they are located in poor or rich areas but the number and kind of committees can be different based on hospital size (Bastani, Vatankhah, & Salehi, 2013). Also, in our study it was found that committees performance in rich and poor areas and in educational and non-educational hospitals don’t have meaningful difference with each other.

It seems at past decades, bases of health care have been weakened and some of analysts believe that the most important point is that an efficient and simple structure at team working prevents acute condition (Shinkman, 2000). Efficient and effective presence of committee members in sessions can increase performance of hospital committees. Regarding the importance of committees in planning and decision-making for current problems as well as preventing future problems, powerful and active committee members can improve committee efficiency (Rate of implemented bills improves). A study of Meraji et al. in Mashhad showed that position of number of held sessions, rate of participants in sessions and distance among sessions were at medium but results in kind of bills and promotion of ethical values were at good level and also in the study of Meraji, presence of representative of university president in ethics committee was 28% that it shows importance of holding hospital committees sessions from view of related managers (Meraji et al., 2014). Our study also shows that besides decreasing the number of hospital committees from 14 committees to 11 committees, the rate of hospital chief attendance in committees sessions in 2012 has been more than 2011 (Table 1).

Our study also showed 58% presences of hospital chief at sessions which indicate the weak presence of higher level managers in committees and this can show managers lack of time and the delegation of managing committees to other people. In this regard, merging committees is offered.

Kind, number and necessity of committees in various countries are different for example in Brazil, presence of hospital infection committee for monitoring hospital infections is necessary but presence of drug and therapy committee is not obligatory and it existed at limited range in hospitals. Data of 2003 show that in 250 governmental and private hospitals in Brazil, only there were 29 drug and therapy committees (Marques & Zucchi, 2006). In Iran, there are 11official committees in hospitals and we observe their number is functionally more than that and sometimes it reaches to 30 committees. Hospital committees are held for hospital goals materialization, help planning, organizing and harmonizing hospital activities and creating active participation ground for all personnel (Standard, 1997). In our study it was shown that about the number of held committees sessions during 2011 and 2012, the most number was related to committee of hospital infection control (113 times), death and side effects of pathology and tissues committee (102 times), drug and therapy committee (89 times) and the least number was related to medical ethics committee (71 times), committee of mother and baby safety and promotion of natural born and feeding with mother milk (79 times) and quality improvement committee (79 times). It seems that the same structure and regulation for all hospitals (governmental, private, specialized, public, small, big, …) as it is now, can’t bring hospital committees efficiency. Instead, flexibility in hospital committees kind, number, session intervals and plans will be more effective.

Decision-making about rate of medical interferences happens at high probability in all health care systems all over the world. Up to now, a little attention has been paid to this question that what role ethical committees can or should play at hospital level? Quantitative and qualitative findings show that remarkable request for ethical consultation was directly expressed by physicians (Stretch, Hurst, & Danis, 2010), but our study shows that among 11 held committees in 2011 and 2012, the least holding was related to ethical committee. Study done by Gaudine et al., as “evolution of medical ethical committees in Canada (2008)” showed that rate of hospitals ethical committee has increased from 58% in1984 to 85% in 2008 (Gaudine et al., 2010).

Another study by Csikai at Pennsylvania as “position of hospitals ethical committees at Pennsylvania (2008)” showed similar results. From 208 studied hospitals, 183 hospitals (88%) had ethical committee (Csikai, 1998). Also in the study of Meraji et al. in Mashhad city, 73% of research population hospitals had ethical committee (Meraji et al., 2014). Also, our study showed that in 2011 only 75% of research population hospitals had ethical committee and in 2012, at 100% research population hospitals this committee sessions was held which shows ascending growth of ethical committee. Study of Ralph in New Zealand as “ethical committee at Oakland hospitals” showed that from 23 studied hospitals, 3 hospitals had ethical committees, 2 hospitals wanted to create these committees and other hospitals expressed problems of their ethical committees at other committees (Pinnock & Crosthwaite, 2004). Also, our study showed that the lowest number of committees held, the lowest bills and the lowest implementation of bills among the 11 committees, was related to ethical committee in 2011 but this three factors improved in 2012.

Rate of bill implementation will promote hospital performance and will bring more job motivation for committee members. It is hoped that all committee bills are implemented but as Table 1 shows, rate of bill implementation is 85% and it has been implemented more in private than governmental hospitals but rate of bills in governmental hospitals in 2011 was three times more than private hospitals which can show higher identification of problems and longer time spent in every session for governmental hospitals. Also, it seems that the high number of bills in each session has caused decreasing the rate of implementing bills. Our offering is that more than 3 bills in each session leads to decreasing implementing bills.

In this research, we have studied the causes of the lack of bill implementation and have found the factors mentioned in table 1. So, by paying attention to these factors, managers should plan for reducing or removing these factors and hold training courses for members and practitioners.

One of important cases in hospital committees is the kind of bills made in committees whether it is related to current problems or predicting future problems (prevention before cure) and the other point is that the subjects relate to which of current hospital resources. In this regard, in a study that was done in Tehran TaminEjtemaei hospitals it was found that prediction of training periods for hospital personnel has a high importance. This study showed that mangers and experts of health care in an environment with high load of job need to have management skills and effective leadership and one of these skills is management of time. It has relationship with promotion of performance quality and it can decrease or delete much amount of managers’ job stress. Also they have expressed that prevention of time waste can lead to improvement of managers’ ability, human resource maintenance, stress decrease and as a result, job satisfaction and mental healthiness increases among managers (Ebrahimi, 2006). Our study showed that around 25% of bills related to future hospital program and especially holding training courses and 75% related to current problems and specially the subjects related to current processes and methods (Table 1). It seems that Current problems being high, prevents planning for future which is itself the cause of not prioritizing plans.

In summary, we can say that the kind of proposed bills in sessions is related to the priority, importance and scope of the bill. This study showed that the most bills are related to current problems which shows existing immediate problems and fewer bills are based on futures and foresight. The most current problems are related to current processes and methods in hospitals. These processes and methods require promotion and these issues are proposed in committees to be analyzed by experts and to be solved.

A study which has been done at medical colleges and general hospital of health ministry in Thailand showed that average of participants number at hospital committees were 14 persons (Panichkul et al., 2011). Also in our study, average of participants number in hospital committees was 85% of total members of each committee. Because of the rate of 84% for the implementation of the bills, it is better that instead of making more bills, more expert time is spent on every bill so that the rate of bill implementation increases.

In a study done by Meraji et.al in hospitals of Mashhad universities, average of hospital committee bills was 4 -5 that is similar to our study (Meraji et al., 2014). Our study shows that average of participants number in hospital committees in 2011 and 2012 is10 to 11 persons which coordinates the offered number of committee members in the most resources.

As we all know, the importance of team working, personnel active participation and presence of hospital chief can promote productivity of hospital committees and a lot of studies have been done at this ground such as Torani et al. (2008) in their study with the title of “effective factors on comprehensive quality implementation” in which they gained these results that rate of personnel participation and rate of paying attention to team working were not at acceptable range and needed boost and continuous improvement (Tourani, Tabibi, & Shahbazi, 2008). Our study also showed that average of committee members participation in sessions was 84% and average presence of hospital chief at committees was 58%. In Meraji et al. study in Mashhad, 72% of sessions were held with all members (6 persons). We can say that attending the boss of committee in the session is one of the effective factors on the way of session holding and making bills because it had been seen that in the time of boss presence, the number of bills and the rate of members cooperation has increased.

According to the role of hospital chief on the improvement of hospital committees performance, our study shows that only in %58 of sessions, hospital chief has attended (in some committees, hospital chief has endowed committee responsibility to one of his deputies). So, for improving the performance of committees, chief presence has an important role and done studies prove the matter that the efficacy of committees in decision-making increases when the chief attends in the session (The number of the bills have been high). For example, Toufighi (2001) in his study gained the result that one of the most important obstacles of widespread quality management is manager's weak support and necessary obligations in processes implementation (Toufighi, 1999).

Rostami in 2002 in his study with title of “crisis factors of widespread quality management” concluded that rate of irregular participation of leaders at strategic committees show their low obligation to this factor (Rostami, 2003) and our study also showed bills usually are not sent to higher levels of hospitals unless for specific items and on the other side, from higher levels of hospitals, feedback from committees performance at province level are not sent to sub hospitals.

As it is shown in Axler study, governmental and private hospitals take into account the processes of re-engineering for the improvement of efficiency and effectiveness, and it was also found in our study that one of the existing problems is related to processes and methods and by designing re-engineering in the structure of hospital committees, we will observe the improvement of hospital committees.

In Hames study, the importance of management committees in innovation is emphasized about which our study also shows that one of the most reasons for lack of bill implementation is lack of exact expert analysis which in turn can be the cause of lack of access to necessary information and knowledge.

Now, from the lowest level in hospital to the university level, obtained information isn’t used enough for the improvement of committees performance and it is used partially.

In Gokenbach study, it is said that committees play an important role in the improvement of productivity that for this purpose, one of the proposed committees by health ministry is quality improvement committee in order for the hospital productivity to be increased.

The weakness of our study was that some of the agenda were handwritten and reading was very difficult. Some of the registered subjects in the agenda were not clear and had ambiguity. On the other hand, because of season random sampling, sometimes the number of sessions was not identical. The other point is that because of the lack of studies related to our subject in recent years, we had to use some studies from several years before.

The strength of our study is the exact studying of the performance of hospital committee sessions during 2 years. The other strength of our study is that we have studied the kind of bills whether it is related to current problems or predicting future problems.

These findings provide some empirical evidence confirming the relationship between committees performance position with hospital productivity. The more the boss presence in committee sessions and the more the track of bills, the better the performance of committee and the more satisfaction of employees. These study findings confirm a number of the findings that have been identified by studies undertaken in some other countries before.

## 5. Conclusion

At general conclusion, we can say that because performance position of each hospital committee and quality of committee sessions can lead to more committee productivity and also committees are tools for distributed knowledge and abilities mixing of persons and various parts of hospital in a form of active and integrated units and they can have effective role in decision-making, managers should recognize sessions results and provide ground for committees improvement. Some suggestions of our study are:

-paying enough attention to regular holding of hospital committees according to previous prediction of Gaunt table, - selection of competent members for every committee,- programming for their active participation;

- holding training courses for members justification about committees duties and instructions, -increasing the implementation of bills and other recognized factors in this study.
